# How Does Our Brain Process Sugars and Non-Nutritive Sweeteners Differently: A Systematic Review on Functional Magnetic Resonance Imaging Studies

**DOI:** 10.3390/nu12103010

**Published:** 2020-09-30

**Authors:** Andy Wai Kan Yeung, Natalie Sui Miu Wong

**Affiliations:** 1 Oral and Maxillofacial Radiology, Applied Oral Sciences and Community Dental Care, Faculty of Dentistry, The University of Hong Kong, Hong Kong, China; 2 Oral and Maxillofacial Surgery, Faculty of Dentistry, The University of Hong Kong, Hong Kong, China; smwong26@hku.hk

**Keywords:** neuroimaging, eating, obesity, sugar, sweetener

## Abstract

This systematic review aimed to reveal the differential brain processing of sugars and sweeteners in humans. Functional magnetic resonance imaging studies published up to 2019 were retrieved from two databases and were included into the review if they evaluated the effects of both sugars and sweeteners on the subjects’ brain responses, during tasting and right after ingestion. Twenty studies fulfilled the inclusion criteria. The number of participants per study ranged from 5 to 42, with a total number of study participants at 396. Seven studies recruited both males and females, 7 were all-female and 6 were all-male. There was no consistent pattern showing that sugar or sweeteners elicited larger brain responses. Commonly involved brain regions were insula/operculum, cingulate and striatum, brainstem, hypothalamus and the ventral tegmental area. Future studies, therefore, should recruit a larger sample size, adopt a standardized fasting duration (preferably 12 h overnight, which is the most common practice and brain responses are larger in the state of hunger), and reported results with familywise-error rate (FWE)-corrected statistics. Every study should report the differential brain activation between sugar and non-nutritive sweetener conditions regardless of the complexity of their experiment design. These measures would enable a meta-analysis, pooling data across studies in a meaningful manner.

## 1. Introduction

Non-nutritive sweeteners were invented as sugar substitutes without calories or with lower calories. With the reduced calorie content, non-nutritive sweeteners should be beneficial to the health of patients with obesity or diabetes mellitus. However, there were conflicting pieces of evidence with regards to how non-nutritive sweeteners affect eating behavior and health.

On the positive side, meta-analyses of human randomized clinical trials have shown that the use of non-nutritive sweeteners could lead to reduced energy intake and body weight [1,2]. On the negative side, exactly the opposite was found in children from epidemiologic studies [3]. Another meta-analysis concluded that sodas sweetened with non-nutritive sweeteners led to an increased risk of obesity compared to sodas sweetened by sugars [4]. One possible explanation for this is that the use of non-nutritive sweeteners induced compositional and functional changes to the intestinal microbiota and hence led to the development of glucose intolerance [5]. However, randomized controlled trials in specific population groups, such as those during gestation, infancy and childhood, were very limited [3,6]. In addition, observational studies and clinical trials that probed into the underlying physiological effects of non-nutritive sweeteners such as glucose metabolism and appetite-regulating hormones were largely heterogeneous in the study designs, resulting in huge confounders [7]. Moreover, an association between the use of non-nutritive sweeteners and increase in body weight was not observed in observational or animal studies [1,2,8].

The unfavorable health outcomes potentially brought on by non-nutritive sweeteners other than weight gain and obesity were also investigated by the literature, such as headaches, depression, behavioral and cognitive effects, cancer, dental caries, diabetes, preterm delivery, and cardiovascular effects [9]. All were without conclusive evidence [9].

The food intake or energy intake behavior is crucial for reducing calorie intake and thus managing weight issue or metabolic syndromes. It was previously demonstrated that humans could sense calorie differences in foods with equal sweetness [10]. The AMP-activated protein kinase was described as a key energy sensor that could modulate the signaling pathways of SIRT1, Ulk1, and mTOR [11]. It is still largely unknown how these signaling pathways relate to the eventual brain responses, but it was reasoned that calorie sensing would eventually be modulated by cerebral processing. Therefore, it would be reasonable to deduce that non-nutritive sweetener and sugar should cause differential activation in the brain, as the former had no or very low calorie content, whereas the latter had a high-calorie content. Understanding the differences in brain response should help researchers and clinicians devise more precise strategies to control the weight of concerned patients, especially concerning the sensitization (increased response) and habituation (reduced response) effects of repeated exposure to the stimuli. However, to the best of the author’s knowledge, there has been no systematic review into differential brain processing of sugars and sweeteners in humans. This systematic review, therefore, aimed to bridge this gap and reveal whether there existed consistent evidence of differential brain processing between the two.

## 2. Materials and Methods

### 2.1. Literature Search and Study Selection

This meta-analysis adhered to the preferred reporting items for systematic reviews and meta-analyses (PRISMA). Three electronic databases, Web of Science (WoS), Scopus, and PubMed, were searched. The search terms followed closely to those used by Nichol et al. [12]. The search strategy involved searching for the following terms in the title and abstract (for WoS) and in the title, abstract and keywords (for Scopus) of the indexed publications: (“non-nutritive sweet*” OR “rebaudioside B” OR “nonnutritive sweet*” OR “non nutritive sweet*” OR “artificial sweet*” OR “natural sweet*” OR “low calorie sweet*” OR “low-calorie sweet*” OR “zero calorie sweet*” OR “zero-calorie sweet*” OR “stevia*” OR “saccharin*” OR “aspartame*” OR “trichlorosucrose*” OR “sucralose*” OR “acetosulfame*” OR “acesulfame*” OR “neotame*” OR “rebaudioside A”) AND (fMRI OR “functional MRI” OR “functional magnet* resonance”). Reference lists of relevant publications were also searched to identify the missed papers. The initial inclusion criteria were all papers identified from these searches and written in English, without restrictions on the types of papers or patient population.

**Figure 1:**
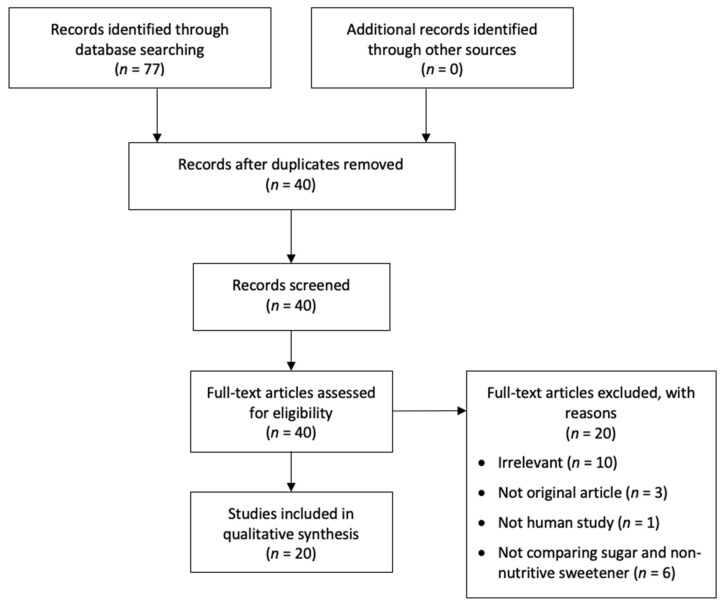
Diagram for literature search.

The search yielded 23 papers from WoS, 34 papers from Scopus, and 20 papers from PubMed. After excluding duplicates, 40 papers remained. The full text of these 40 papers were evaluated to exclude those that were: (i) irrelevant; (ii) not original articles; (iii) not human studies; or (iv) not comparing sugar and non-nutritive sweetener. Two independent reviewers (AY and NW) did the screening. Disagreements were resolved by discussion and reaching consensus. Finally, 20 studies remained (Figure 1).

### 2.2. Data Extracted from the Analyzed Studies

Two independent reviewers (A.Y. and N.W.) extracted the following data from each paper: authors, publication year, journal, participants’ characteristics (including the age, gender, body mass index (BMI), and medical condition), the sugar and non-nutritive sweetener used, duration of fasting before experiment, the tasks of the fMRI study, the statistical threshold used for fMRI data analysis, and whether sugars or non-nutritive sweeteners caused a larger brain response and where. Disagreements were resolved by discussion and reaching consensus.

### 2.3. Study Quality Assessment

The quality of the studies was assessed with seven criteria, adapted from the criteria used by Nichol et al. [12], namely: (i) Was the research question clearly stated? (ii) Were the inclusion and exclusion criteria clearly stated? (iii) Were study participants’ BMI clearly reported? (iv) Was a power analysis conducted to calculate the required sample size? (v) Was the dropout rate or data exclusion rate 20% or lower? (vi) Was the population referenced in the conclusion appropriate? (vii) Were the participants controlled for food and drink ingestion before the study? Please note that the last criterion here was different from that of Nichol et al. [12], who asked if there were 20 or more participants that received sweetener without additional caloric intake, which was not useful in this review as fMRI studies usually have few participants and was thus replaced.

## 3. Results

### Study Characteristics

There were 20 studies being reviewed, published between 2005 and 2019, in journals with an impact factor. The number of participants per study ranged from 5 to 42 (Table 1), with a total number of study participants of 396. Seven studies recruited both males and females, 7 were all-female and 6 were all-male. Across studies, the mean age of the participants ranged from 20.4 to 50.9 years. The mean BMI ranged from 21.5 to 29.6 kg/m^2^. Participants with obesity were involved in 5 studies, eating disorders in 2, and schizophrenia in 1. There were 11 studies that recorded the brain activity concerning the tasting of sweet solutions, 5 concerning tasks after pre-loading with sweet solutions, and 4 concerning resting condition after pre-loading with sweet solutions. In terms of non-nutritive sweeteners, sucralose was involved in 7 studies, saccharin in 7, aspartame in 5, acesulfame in 3, stevia in 2, allulose in 1, and cyclamate in 1. Due to the heterogeneity of the studies and the fact that only 5 studies reported brain regions with significant results from whole-brain analysis in standardized brain coordinates, a coordinate-based meta-analysis to evaluate the differential brain responses elicited between sugar and non-nutritive sweetener was not performed.

The 20 studies scored from 4 to 12 out of a maximum of 14 (Table 2). Most studies scored 10 or 12, indicating a high quality. All studies stated the research question clearly, whereas most studies had a dropout rate of 20% or lower and controlled for food and drink before the experiment. On the contrary, only one study (5%) performed a power analysis to calculate the required sample size and reported that their sample was underpowered. Meanwhile, the appropriateness of the population referenced in the conclusion was mixed. This was because studies recruiting a single sex sample often did not explicitly remind readers about it in the conclusion paragraph.

## 4. Discussion

### 4.1. Differential Brain Responses during Tasting

Three studies have reported larger brain responses elicited by sugars than sweeteners among the generally healthy subjects, such as in the insula/operculum [16,19], cingulate [16,19], striatum [16], orbitofrontal cortex [15], superior frontal gyrus [16], and precentral gyrus [19]. No study reported vice versa for generally healthy subjects. One study reported no significant difference between sugars and sweeteners [31].

For specific subject groups, the involved brain regions were different. For patients recovered from bulimia, sugars elicited larger responses than sweeteners in the insula/operculum and striatum [23]. Besides, two studies reported sweeteners triggering larger responses than sugars, namely the orbitofrontal cortex among non-diet soda drinkers [18], and the insula/operculum and striatum among patients recovered from anorexia [23].

Repeated exposure to sugars and sweeteners could affect the brain responses, as the brain would be sensitized to the stimuli. The extent of the sensitization was different between the two. Among the healthy subjects and patients recovered from bulimia, the increase in the response level to sugars was larger than sweeteners in the striatum and precuneus [19,32], cingulate, thalamus, and cerebellum [32]. In contrast, the increase in the response level to sweeteners was larger than sugars in these brain regions among patients recovered from anorexia [32].

One study reported an interaction between stimulus type and appetite [31]. Several brain regions responded differentially between sugars and sweeteners in hungry and satiated conditions, including the insula/operculum, cingulate, superior frontal gyrus, middle frontal gyrus, inferior frontal gyrus, precentral gyrus, postcentral gyrus, thalamus, superior temporal gyrus, middle temporal gyrus, inferior temporal gyrus, fusiform gyrus, and inferior parietal gyrus [31]. Haase et al. [20] did reported the differential responses between hungry and satiated conditions, but with sugars and sweeteners considered separately, so there were no directly comparable results.

Four studies reported results from sugars and sweeteners separately [13,17,20,21]. Two of them concluded that sugars elicited responses in more brain regions than sweeteners. Chambers et al. [13] reported that both activated the insula/operculum and dorsolateral prefrontal cortex, with sugars additionally activated the striatum and cingulate. Meanwhile, Haase et al. [20] reported that both activated the striatum, thalamus, cuneus, parahippocampus, and hippocampus. Sugars additionally activated the insula/operculum, cingulate, amygdala, orbitofrontal cortex, middle frontal gyrus, medial frontal gyrus, postcentral gyrus, ventral tegmental area/substantia nigra, hypothalamus, superior temporal gyrus, fusiform gyrus, precuneus, cerebellum, and angular gyrus. Sweeteners did not activate these brain regions, but the precentral gyrus and lingual gyrus instead.

The most common brain regions reported in the above studies seemed to be the insula/operculum (7 studies), cingulate (7), and striatum (6). They were frequently reported in meta-analyses of food and taste neuroimaging studies as the core structures of a taste processing network [33,34], and especially for processing the hedonic values of food [34]. Indeed, when viewing high-caloric food pictures, women with obesity had a higher activity level in the striatum than women with normal-weight, and the activity levels of striatum, insula, and cingulate were positively correlated to the body mass index [35]. The phenomenon of higher activation in the group with obesity was also observed in men, even after eating [36]. Moreover, the activity level of the cingulate and striatum reduced in response to high-caloric food pictures after gastric bypass surgery to control obesity [37]. The differential activations of these brain regions by sugars and sweeteners offered a glimpse into the underlying different neural processing of them. However, there was no clear consensus whether sugars or sweeteners elicited larger responses in the brain, though results tended to suggest that the former group would lead to larger or more widespread brain responses.

### 4.2. Differential Brain Responses during Tasks after Pre-Loaded with Sweet Beverages

Five studies investigated the differential effect of ingesting beverages with sugars and sweeteners on the brain responses to performing tasks subsequently [14,24,26,27,28]. Compared with subjects who were lean, subjects with obesity had larger brain responses when viewing food pictures after being pre-loaded with sugar than sweetener in the insula/operculum, cingulate, amygdala, hippocampus, and visual cortex [14]. Healthy subjects also had larger brain responses in the superior parietal gyrus when viewing general pictures after being pre-loaded with sugar compared to sweetener [24]. They also had larger responses in the hippocampus, precuneus, supramarginal gyrus, paracentral lobule, middle frontal gyrus, and inferior parietal gyrus when recalling the memory of pictures after being pre-loaded with sugar compared to sweetener [24]. A larger response was observed in the insula/operculum, precentral gyrus, postcentral gyrus, and inferior temporal gyrus after being pre-loaded with sweetener rather than sugar [24]. Compared with pre-loading with sugar, tasting sweet solutions after pre-loading with sweetener would have larger brain responses in the insula/operculum, inferior frontal gyrus, and inferior parietal gyrus. Word encoding was also investigated. For patients with schizophrenia, word encoding would result in larger brain responses after being pre-loaded with sugar rather than sweetener, in the dorsolateral prefrontal cortex, parahippocampus, cuneus, and inferior temporal gyrus [27]. Finally, pre-loading with sugar would cause a larger response in the hippocampus than sweetener when healthy subjects performed an arithmetic task [28]. These examples showed that there was no simple phenomenon of whether ingesting sugar or sweetener would heighten the brain activity level when tasks were performed.

### 4.3. Differential Brain Activity Levels at Resting State after Pre-Loaded with Sweet Beverages

Four studies investigated the effects of ingesting beverages with sugars and sweeteners on the brain activity level at rest [22,25,29,30]. Kilpatrick et al. [22] found that there was a larger reductive effect of brain activity levels by ingesting sugars than sweeteners in the brainstem regions, such as the trigeminal nucleus, locus coeruleus, periaqueductal grey, and reticular nucleus. Sweeteners had a larger reductive effect in the nucleus tractus solitaries [22]. Though no significant differences in the functional connectivity were found between sugar and sweetener, across the conditions, subjects with obesity had a greater connectivity between the right lateral hypothalamus and a reward-related brain region, and weaker connectivity with homeostasis and gustatory-related brain regions than subjects who were lean [22]. Van Opstal et al. [29] found that sugars reduced the activity level in the cingulate, ventral tegmental area, insula, lingual gyrus, fusiform gyrus, and striatum, whereas sweeteners had no effect. Besides, sucralose significantly increased the eigen vector centrality values in the cingulate, central gyri, and temporal lobe [29]. Another van Opstal et al. [30] study found that sugars reduced the activity level in the hypothalamus profoundly, whereas sweeteners reduced it much more mildly. In this study, they found that sugars increased the activity level shortly in the ventral tegmental area, whereas sweeteners increased it continuously [30]. Therefore, results from these two studies were a bit contradictory to each other in terms of the effect on the activity level in the ventral tegmental area. Meanwhile, Smeets et al. [25] also reported a prolonged reduced activity level in the hypothalamus caused by sugars but not by sweeteners. The brainstem, hypothalamus, and ventral tegmental area are targeted by various hormones and neural signals and are thus highly related to the processing of homeostatic energy balance, which is regulated in the long-term by leptin and insulin and in the short-term by circulating gastrointestinal hormones such as ghrelin, glucagon-like peptide 1 and peptide tyrosine [38]. There exists a complex energy-balance-regulating neural circuit that consists of the pituitary gland, brainstem, periaqueductal grey, thalamus, and various nuclei of the hypothalamus, and a key component of it is the melanocortin system in the arcuate nucleus of the hypothalamus [39]. Appetite is strongly modulated via the interplay between neuropeptide Y and proopiomelanocortin [39]. Therefore, it was reasonable to see that most studies reported an ingestion of energy-carrying sugary solutions led to a significant change in the activity level of these relevant regions.

### 4.4. Limitations of This Study and Future Perspectives

Many of the reviewed studies had small sample sizes, or reported uncorrected or false-discovery rate (FDR)-corrected statistics, which might be too liberal and lead to an increased false positive rate [40]. Some studies did not report the simple differential brain activation between sugar and non-nutritive sweetener (sugar > sweetener and vice versa), which would be crucial for conducting a meta-analysis. Moreover, non-fMRI studies, such as an EEG study by Crézé et al. [41], were omitted from this work. Readers should also be aware that the studies adopted different fasting durations, which further complicated any potential between-study comparisons, as longer fasts were associated with higher activity in the cerebellum, thalamus, and striatum in response to sweet taste [42]. Future studies, therefore, should recruit a larger sample size, adopt a standardized fasting duration (preferably 12 h overnight, which is the most common practice and brain responses are larger in the state of hunger), and reported results with familywise-error rate (FWE)-corrected statistics. Every study should report the differential brain activation between sugar and non-nutritive sweetener conditions regardless of the complexity of their experiment design. These measures would enable a meta-analysis pooling data across studies in a meaningful manner. In addition, future studies are recommended to recruit both males and females, as a recent review by Yunker et al. [43] also pointed out that many neuroimaging studies of NNS recruited same-sex cohorts only. That review also concluded that the differential brain responses elicited by NNS and caloric sweeteners did not seem to relate to metabolic findings [43]. As mentioned in the Introduction, humans could sense calorie differences in foods with equal sweetness [10] and that there are many relevant underlying signaling pathways such as those of SIRT1, Ulk1, and mTOR [11]. Therefore, future fMRI studies should also collect the metabolic data of subjects, such as changes in insulin levels.

## 5. Conclusions

This systematic review highlighted that there were few fMRI studies evaluating the differential cerebral processing of sugars and non-nutritive sweeteners, and their study designs were largely varied. The existence of inter-individual differences in response to sugar and non-nutritive sweeteners might also lead to the inconsistency and such as factors influencing individual differences should further be investigated. There was no consistent pattern suggesting that sugar or sweetener elicited larger brain responses. However, the brain regions often reported among these studies were the insula/operculum, cingulate, striatum, brainstem, hypothalamus, and ventral tegmental areas. They were related to taste processing, hedonic evaluations of food, and the processing of homeostatic energy balance. It should be noted that eight studies (40%) recruited fewer than 16 participants, a rudimentary threshold of recommended sample size for detecting the moderate effect size of fMRI findings [44], and hence their results might not be readily reproduced by future studies. With the large number of sugars and chemically distinctive sweeteners being consumed in our daily meals, more studies should be conducted as soon as possible to investigate their neural correlates, so that clinicians can further devise strategies to manage patients who require reduced sugar intake.

## Figures and Tables

**Table 1:** Details of the 20 analyzed studies.

Study	Journal (2018 Impact Factor)	Sample Size	Age, Mean ± SD	BMI ± SD	Medical Condition Involved	Sugar Used	Non-Nutritive Sweetener Used	Fasting before Experiment	Task of fMRI	Any Statistical Tests to Directly Compare Brain Responses to Sugar and Sweetener	Statistical Threshold ^a,b^	Main Findings
Chambers et al. 2009 [13]	J Physiol-London (4.984)	8 (8M)	29 ± 9	23.8 ± 2.5	Healthy	Glucose	Saccharin	Overnight	Passive tasting of the sweet solutions	No (separate tests against baseline)	*p* < 0.05, FWE corrected	Sugar caused larger brain responses in anterior cingulate and striatum
Connolly et al. 2013 [14]	Neurogastroenterol Motil (3.803)	20 (20F)	25.6, range = 18–40	27.7, range = 19–37	Obesity	Sucrose	Truvia (stevia-based)	6 h	Reported brain responses to viewing food images after drinking sweet beverages	Yes	Clusters with a peak z >3.30 and >60 voxels	Sugar and sweetener engaged similar brain regions. Females with obesity had larger brain responses than lean females for sugar but not sweetener condition in anterior cingulate, anterior insula, amygdala and hippocampus
Di Salle et al. 2013 [15]	Gastroenterology (19.809)	9 (5M, 4F)	23 ± NA	NA	Healthy	Sucrose	Aspartame + acesulfame	Unclear	Passive tasting of the sweet solutions	Yes	*p* < 0.05, corrected with unknown method	Sugar and sweetener caused increased responses in different brain regions. With carbonation, the differential responses largely diminished
Frank et al. 2008 [16]	NeuroImage (5.812)	12 (12F)	27 ± 6	22 ± 2	Healthy	Sucrose	Sucralose	Overnight	Passive tasting of the sweet solutions	Yes	*p* < 0.05 with clusters >8 voxels, uncorrected	Sugar caused larger brain responses in anterior insula, anterior cingulate, caudate, and superior frontal gyrus. Sugar engaged dopaminergic midbrain regions but not sweetener
Gramling et al. 2019 [17]	Nutrients (4.171)	28 (12M, 16F)	50.9 ± 17.4	29.6 ± 6.5	Obesity	Sucrose	Saccharin	12 h	Tasting of sweet solutions and evaluated the pleasantness	No (separate tests against baseline)	*p* < 0.015, FWE corrected	Sugar caused greater responses in memory and reward regions. Sweetener caused greater responses in memory and information processing regions
Green and Murphy 2012 [18]	Physiol Behav (2.635)	24 (10M, 14F)	23.5 ± 2.8	26.1 ± 5.9	Obesity	Sucrose	Saccharin	12 h	Tasting of sweet solutions and evaluated the pleasantness	Yes	*p* < 0.01, FWE corrected	Sweetener caused greater responses than sugar in non-diet soda drinkers in orbitofrontal cortex. For diet soda drinkers, there was no difference
Griffioen-Roose et al. 2013 [19]	PLOS One (2.776)	40 (15M, 25F)	21 ± 2	21.5 ± 1.7	Healthy	Sucrose	Sucralose + acesulfame	3 h	Tasting of sweet solutions and evaluated the pleasantness	Yes	*p* < 0.05 with clusters >8 voxels, uncorrected	Sugar caused larger brain responses in Rolandic operculum, precentral gyrus and middle cingulate
Haase et al. 2009 [20]	NeuroImage (5.812)	18 (9M, 9F)	20.7 ± 1.0	23.7 ± NA	Healthy	Sucrose	Saccharin	12 h	Passive tasting of sweet solutions	No (separate tests against baseline)	*p* < 0.0005, FWE corrected	Sugar elicited responses in more brain regions
James et al. 2009 [21]	NeuroReport (1.146)	9 (6M, 3F)	29 ± 4.3	NA	Healthy	Sucrose	Aspartame	Unclear	Passive tasting of sweet solutions	No (separate tests against time)	*p* < 0.05, uncorrected	Sweetener elicited brain responses of longer duration in the insula
Kilpatrick et al. 2014 [22]	Gastroenterology (19.809)	22 (22F)	26.3 ± 1.6	27.6 ± 0.6	Obesity	Sucrose	Truvia (stevia-based)	6 h	Reported brain activity at resting state after drinking sweet beverages	Yes	*p* < 0.05, FWE corrected	Sugar and sweetener caused increased responses in different brain regions
Oberndorfer et al. 2013 [23]	Am J Psychiatry (13.655)	42 (42F)	40.7 ± 4.2	22.3 ± 2.1	Anorexia and bulimia	Sucrose	Sucralose	Overnight	Passive tasting of sweet solutions	Yes	*p* < 0.005 with clusters >32 voxels, FWE corrected	Sugar caused larger brain responses in patients recovered from bulimia. Sweetener caused larger responses in patients recovered from anorexia
Parent et al. 2011 [24]	Neuropsychologia (2.872)	14 (14M)	24.1, range = 19–34	NA	Healthy	Glucose	Saccharin	Overnight	Reported brain activity at viewing pictures and recalling them, after drinking sweet solutions	Yes	*p* < 0.001 with clusters >2 voxels, uncorrected	Sugar caused larger widespread brain responses and connectivity
Smeets et al. 2005 [25]	Am J Clin Nutr (6.568)	5 (5M)	20.4 ± 5.6	21.7 ± 2.5	Healthy	Glucose	Aspartame	Overnight	Reported brain activity at resting state after drinking sweet beverages	No (separate tests against time)	*p* = 0.0018, Bonferroni corrected	Sugar elicited prolonged decreased brain responses but not sweetener
Smeets et al. 2011 [26]	NeuroImage (5.812)	10 (10M)	23.3 ± 2.8	22.4 ± 2.0	Healthy	Sucrose	Aspartame + acesulfame K + cyclamate + saccharin	2 h	Reported brain activity at passive tasting of sweet beverages, before and after drinking sweet beverages	Yes	*p* < 0.005, uncorrected	Sugar and sweetener caused larger responses in different brain regions. The differential responses were modulated by pre-loading of sweet beverages
Stone et al. 2005 [27]	Neurobiol Learn Mem (3.010)	8 (5M, 3F)	38.8 ± 10.7	28.6 ± 4.9	Schizophrenia	Glucose	Saccharin	8 h	Reported brain activity at verbal encoding task after drinking sweet beverages	Yes	*p* < 0.005 with clusters >5 voxels, uncorrected	Sugar caused larger brain responses in parahippocampus
Tyron et al. 2015 [28]	J Clin Endocrinol Metab (5.605)	19 (19F)	26.9 ± 6.5	25.7 ± 3.3	Obesity	Sucrose	Aspartame	Unclear	Reported brain activity at stress task, after drinking sweet beverages for 2 weeks	Yes	*p* < 0.05, FDR corrected	Sugar treatment caused larger brain responses in hippocampus
Van Opstal et al. 2019a [29]	Nutr Neurosci (3.950)	20 (20M)	22.2 ± 1.3	22.4 ± 1.1	Healthy	Glucose, fructose	Sucralose, allulose	Overnight	Reported brain activity at resting state before and after drinking sweet beverages	No	*p* < 0.05, FWE corrected	Sugar caused decreased brain activity in cingulate, insula and basal ganglia
Van Opstal et al. 2019b [30]	Nutrition (3.591)	16 (16M)	22.4 ± 1.3	22 ± 1.2	Healthy	Glucose, fructose, sucrose	Sucralose	10 h	Reported brain activity at resting state after drinking sweet beverages	No	*p* < 0.05, uncorrected	Sugar caused more decreased brain activity in hypothalamus. Sweetener caused more increased brain activity in the ventral tegmental area
Van Rijn et al. 2015 [31]	Behav Brain Res (2.770)	30 (30F)	22 ± 3	22.6 ± 1.4	Healthy	Maltodextrin + Sucralose (sweet with energy)	Sucralose (sweet without energy)	3 h	Passive tasting of sweet solutions under hungry and satiated conditions	Yes	*p* < 0.001 with clusters >8 voxels, uncorrected	In overall, sugar and sweetener did not have significant difference. However, sugar caused larger differential brain response between hunger and satiety states
Wagner et al. 2015 [32]	Psychiatry Res Neuroimaging (2.270)	42 (42F)	26.7 ± 6.0	21.9 ± 2.1	Anorexia and bulimia	Sucrose	Sucralose	Overnight	Passive tasting of sweet solutions	Yes	*p* < 0.05 with clusters >30 voxels, FWE corrected	Sugar caused larger brain response upon repeated exposure in patients recovered from bulimia and healthy controls. Sucralose caused larger brain response upon repeated exposure in patients recovered from anorexia

^a^ FDR, false discovery rate. ^b^ FWE, familywise error rate. NA—Not applicable.

**Table 2:** Quality assessment.

Study	Criterion							Total
	1	2	3	4	5	6	7	Score
Chambers et al. 2009 [13]	2	2	2	0	2	2	2	12
Connolly et al. 2013 [14]	2	2	2	0	2	2	2	12
Di Salle et al. 2013 [15]	2	2	0	0	2	2	0	8
Frank et al. 2008 [16]	2	2	2	0	2	0	2	8
Gramling et al. 2019 [17]	2	2	2	0	2	2	2	12
Green and Murphy 2012 [18]	2	2	2	0	2	2	2	12
Griffioen-Roose et al. 2013 [19]	2	2	2	0	2	2	2	12
Haase et al. 2009 [20]	2	2	2	0	2	2	2	12
James et al. 2009 [21]	2	0	0	0	0	2	0	4
Kilpatrick et al. 2014 [22]	2	2	2	0	2	2	2	12
Oberndorfer et al. 2013 [23]	2	2	2	0	2	2	2	12
Parent et al. 2011 [24]	2	2	0	0	2	2	2	10
Smeets et al. 2005 [25]	2	2	2	0	2	0	2	10
Smeets et al. 2011 [26]	2	2	2	0	2	0	2	10
Stone et al. 2005 [27]	2	2	2	0	2	2	2	12
Tyron et al. 2015 [28]	2	2	2	0	2	0	2	10
Van Opstal et al. 2019a [29]	2	2	2	2	2	0	2	12
Van Opstal et al. 2019b [30]	2	2	2	0	2	0	2	10
Van Rijn et al. 2015 [31]	2	2	2	0	2	0	2	10
Wagner et al. 2015 [32]	2	2	2	0	2	2	2	12

Scores for each criterion range from 0 to 2, with 0 being not reported or not met, 1 being partially met, and 2 being completely met. Thus, the total score ranges from 0 to 14. The criteria were as follows: (1) Was the research question clearly stated? (2) Were the inclusion and exclusion criteria clearly stated? (3) Were study participants’ BMIs clearly reported? (4) Was a power analysis conducted to calculate the required sample size? (5) Was the dropout rate or data exclusion rate 20% or lower? (6) Was the population referenced in the conclusion appropriate? (7) Were the participants controlled for food and drink ingestion before the study?
